# Spatiotemporal Transmission and Determinants of Typhoid and Paratyphoid Fever in Hongta District, Yunnan Province, China

**DOI:** 10.1371/journal.pntd.0002112

**Published:** 2013-03-14

**Authors:** Jin-Feng Wang, Yan Wang, Jing Zhang, George Christakos, Jun-Ling Sun, Xin Liu, Lin Lu, Xiao-Qing Fu, Yu-Qiong Shi, Xue-Mei Li

**Affiliations:** 1 State Key Laboratory of Resources and Environmental Information System, Institute of Geographic Sciences and Natural Resources Research, Chinese Academy of Sciences, Beijing, China; 2 Chinese Center for Disease Control and Prevention, Beijing, China; 3 Department of Geography, San Diego State University, San Diego, California, United States of America, and College of Environment & Natural Resources, Zhejiang University, Hangzhou, China; 4 Yunnan Provincial Center for Disease Control and Prevention, Kunming, Yunnan, China; 5 Center for Disease Control and Prevention of Hongta District, Yuxi, Yunnan, China; University of California San Diego School of Medicine, United States of America

## Abstract

**Background:**

Typhoid and paratyphoid fever are endemic in Hongta District and their prevalence, at 113 per 100,000 individuals, remains the highest in China. However, the exact sources of the disease and its main epidemiological characteristics have not yet been clearly identified.

**Methods and Findings:**

Numbers of typhoid and paratyphoid cases per day during the period 2006 to 2010 were obtained from the Chinese Center of Disease Control (CDC). A number of suspected disease determinants (or their proxies), were considered for use in spatiotemporal analysis: these included locations of discharge canals and food markets, as well as socio-economic and environmental factors. Results showed that disease prevalence was spatially clustered with clusters decreasing with increasing distance from markets and discharge canals. More than half of the spatial variance could be explained by a combination of economic conditions and availability of health facilities. Temporal prevalence fluctuations were positively associated with the monthly precipitation series. Polluted hospital and residential wastewater was being discharged into rainwater canals. *Salmonella* bacteria were found in canal water, on farmland and on vegetables sold in markets.

**Conclusion:**

Disease transmission in Hongta district is driven principally by two spatiotemporally coupled cycles: one involving seasonal variations and the other the distribution of polluted farmland (where vegetables are grown and sold in markets). Disease transmission was exacerbated by the fact that rainwater canals were being used for disposal of polluted waste from hospitals and residential areas. Social factors and their interactions also played a significant role in disease transmission.

## Author Summary

Typhoid and paratyphoid epidemics are serious events in low-income countries; these diseases are notorious for their high infection rate, long duration, and heavy health burden. In China, typhoid and paratyphoid are considered to be under control, although the situation varies considerably from place to place. During 2010 the disease incidence was 1.2 per 100,000 at the national level. The highest incidence, 113 per 100,000, occurred in the Hongta District of Yunnan province, in southwestern China. We used quantitative spatiotemporal analysis to explore the relationship between the incidence of these diseases and a number of factors suspected of influencing their occurrence. We found that cases tended to occur near discharge canals and polluted farmland where vegetables are grown for sale in local markets. The spatial characteristics of disease transmission were associated with the seasonal variations common to all intestinal infectious diseases. The findings of this work could inform local public health planners and the health directorate and help to improve public health intervention programs in regions with the highest incidence of these diseases.

## Introduction

Typhoid and paratyphoid fever are serious infections, particularly in low-income countries, causing about 16 million cases and 600,000 deaths annually worldwide [1]. These diseases are notorious for their high infection rate, long duration, and heavy health burden. In China, typhoid and paratyphoid have been recorded daily since 2004 by the National Infectious Diseases Reporting Information System (NIDRIS) of the Chinese CDC, which enables all health care institutes across the country to report individual case information of important infectious diseases in real time using the Internet. Typhoid and paratyphoid fever are now regarded as being under control nationwide, and during 2010 the national disease incidence was 1.2 per 100,000.However, prevalence, varied considerably from place to place with the highest incidence of 113 per 100,000,occurringinHongta district of Yunnan province in southwestern China [2], [3].

Water and food sanitation and environmental awareness generally can effectively reduce food and water borne diseases. A total of 3538 cases of typhoid and paratyphoid fever were reported in Hongta district during the period 2006–2010. Sanitation conditions in general are better for Hongta residents than for people in other parts of the province: a previous investigation showed that 50% of the population did not drink unboiled water, 91% washed their hands before dinner and 79% washed fruit before eating it. The case-control study [4], which included80 pairs of cases and controls, showed that adding fresh mint (OR = 2.17, 95%CL: 1.04–4.54) to breakfast, eating uncooked vegetables (OR = 2.29, 95%CL: 1.24–4.24) at restaurants or roadside food sites, and eating flavoring that contained fresh caraway and mint (OR = 2.38, 95%CL: 1.00–5.69) are all risk factors for typhoid and paratyphoid fever in the Hongta district. The incidence reaches its highest peak during the June–October period, and then decreases, reaching its lowest value during February. This pattern is consistent with the seasonal characteristics of other intestinal infectious diseases. During the high incidence period the weather is hot and humid, allowing more rapid bacteria reproduction; also during this weather people often eat more raw and cold food. The cycle has been repeated for many years and the situation has become chronic. Furthermore, in the Hongta district, infection is often left untreated and only 42.6%–47.9% of carriers receive medical care. This leads to a large number of infection sources and carriers, and contributes significantly to the persistence of these diseases in the region.

In recent years, intervention has become a crucial determinant of disease transmission [5]–[7]. During 2008, the typhoid and paratyphoid fever control program (2008–2010) in the Hongta district focused on the restriction of fresh caraway and mint eating in restaurants and roadside food sites, the surveillance and hospital treatment of patients and carriers using appropriate antibiotics, improvement of the environment, health education, food hygiene(especially for fresh vegetables), drinking water disinfection and vaccination. As a result, the morbidity in the Hongta district dropped from 232 per100,000 during 2000–2007 to 113 per 100,000 in 2010, a decrease of about 51%, but still high compared with other regions of China.

Intervention would be much more efficient if the disease sources, transmission paths and factors were accurately identified. According to other studies [8]–[16], and our present work, the following disease transmission hypotheses are proposed: (1) Polluted water from hospitals and residential sewage discharged into canals is an important disease determinant. (2) Polluted vegetables sold in the markets constitute another determinant. (3) Social and environmental factors interact together to influence disease transmission.

The environmental health processes and variables involved in the above hypotheses are distributed across space and time [17]. Understanding this pattern should provide valuable clues about disease sources and determinants. The disease pattern can be rigorously addressed using stochastic spatiotemporal analysis [18]–[20] and powerful GIS technology [21]–[25] available at a relatively lower cost than the mainstream epidemiological techniques. A non-random disease distribution will always display spatial disease clusters [22]. The concordance between the spatial pattern of a disease and that of a contributing factor or determinant usually indicates the power of that determinant [23], [24]. Integration of GIS with statistical models of disease distribution forms a nationwide web-based automated system for early disease outbreak detection and rapid response in China [25]. The dataset and tools have already been used in the analysis of typhoid transmission [26]–[28]. The main objective of the present study is to identify the sources, transmission processes, and determinants of typhoid and paratyphoid fever in the Hongta District, based on the space-time analysis of the available epidemiological and notification data.

## Methods

Data for the typhoid and paratyphoid cases for the Hongta district, and the factors associated with it were collected and a conceptual model was developed to study the proposed transmission hypotheses based on the available data. Transmission sources and population exposure were studied using geographical detectors and epidemiological surveys.

### Study Area

**Figure 1:**
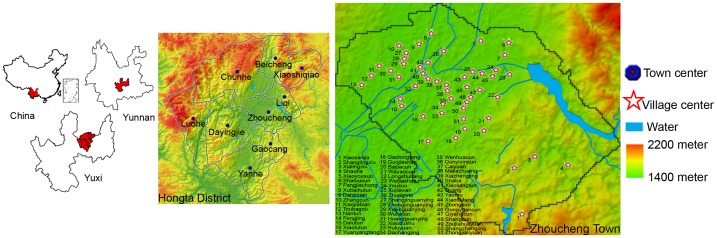
Hongta district and Zhoucheng town. Background color denotes elevation variation.

The Hongta district is located in the center of the Yunnan province, with coordinates 24°08′N–24°32′N, 102°17′E–102°41′E, at an elevation of 1630 m, (see Figure 1). The district's climate is subtropical and sub-humid. The total area is 1004 km^2^, 85% of which is mountains and the rest is irrigated farmland. The total population is 420,553, of which 268,635 are rural people. There are 11 towns and 81 administrative villages. The per capita income of the farmers is 4432 CNY, which lies in the middle of the range of farmers' income country-wide.

### Data Sources

Data on incidence included both probable and laboratory-confirmed cases as defined by the national *Salmonella typhi* and paratyphoid standards. Illness caused by *S.typhi* is often characterized by insidious onset of sustained fever, headache, malaise, anorexia, relative bradycardia, constipation or diarrhea, and non-productive cough. Laboratory criteria for diagnosis are based on positive isolation of *S. typhi* or *paratyphi* in blood, stool, or other clinical specimen. A confirmed case is defined as a clinically compatible case that is laboratory-confirmed. It was estimated that the proportion of unreported cases was below 5%. These cases generally concerned mild illnesses identified in both hospitals and community. Daily numbers of confirmed typhoid and paratyphoid cases during the period 2006–2010 were obtained from the national infectious diseases reporting information system of the Chinese CDC. The system covers 95% of the country's population and provides certain patient information, including personal identification number (ID), specimen used for testing paratyphoid pathogen, and family address defined by GPS. Typhoid and paratyphoid cases were diagnosed according to clinical symptoms and blood culture Salmonella-positive test (the national diagnostic criteria for typhoid and paratyphoid fever were used [29]). All study data were stripped of personal information.

Demographic and socio-economic data were obtained from the local Statistics Bureau and included GDP per capita, number of medical institutions, population, percentage of farmers in the population, and geographical distribution of the villages.

Annual precipitation and other meteorological data were available from the China meteorological data-sharing network, and were interpolated using the spatial kriging technique to cover the entire study area.

Geomorphic data were obtained from the China geomorphy map (1∶1,000,000 scale), and seven categories were defined: plain, terrace, hill, low-relief mountain, middle-relief mountain, high-relief mountain and extremely high-relief mountain. Geomorphic data were provided by the state key Laboratory of Resources and Environmental Information Systems from the Institute of Geographic Sciences and Natural Resources Research, Chinese Academy of Science.

The Normalized Difference Vegetation Index (NDVI), acquired by satellite remote sensing imaging, reflects greenness of vegetation, photosynthetic rate, and vegetation metabolic intensity and measures seasonal and inter-annual changes. This makes NDVI an efficient indicator of vegetation and eco-environment in the locations where typhoid/paratyphoid Salmonellae occur. We used monthly NDVI values with spatial resolution 1 km by 1 km.

The spatial distribution of canals, markets, water wells, streets, and landuse in Zhoucheng town (Hongta district) were provided by the Yuxi city Bureau of Mapping and Survey. Field surveying showed that hospital disposal and residential sewage are connected and discharged into the rainwater canal system.

Water and vegetable samples were collected from markets and farmland, canals irrigating the farmland, hospital disposal products and residential sewage. The samples were tested by pulsed field gel electrophoresis, a technique used for the separation of and identification of Salmonella DNA.

The data were organized either into excel tables or handled by means of ENVI and ArcGIS 10.1 software.

### Conceptual Model

**Figure 2:**
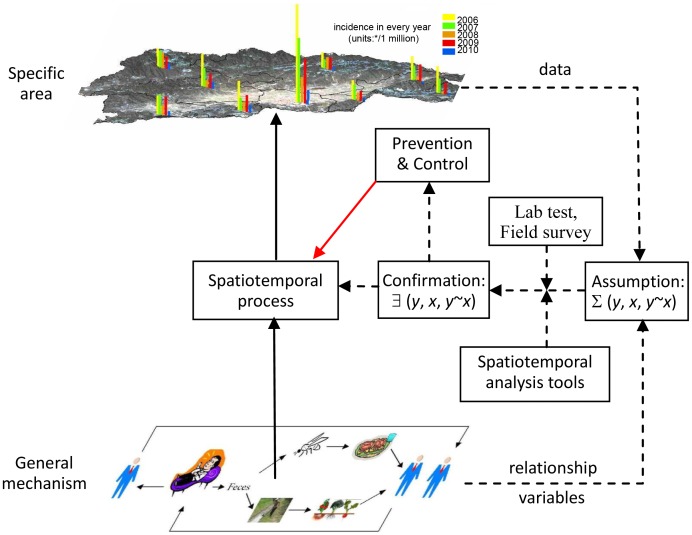
Conceptual model. The top half of the diagram shows the observed yearly disease incidence. The bottom half of the diagram illustrates the infected patient (patients lying on beds to the left) and the route of infected excretion products via flies or polluted irrigation water and infected vegetables to susceptible individuals (standing persons on right) who become further infected individuals (lying on beds). Disease transmission mechanisms (bottom part of image) and observations (top) suggest study hypotheses involving disease sources and factors (*x*), transmissions (*y*∼*x*), and spatiotemporal patterns (*y*).

Figure 2 outlines the study design. The observed yearly disease incidence for towns located in the Hongta district is shown in the top part of the diagram. The bottom part of the diagram depicts the infected patient and the route taken by the patient's unsterilized excretion products to water sources that are then used to irrigate vegetables grown on the farmland. Transmission to susceptible patients may occur either directly via flies or polluted water or via vegetables grown on the polluted farmland. Infected individuals may recover and become susceptible, and then become infected again. Untreated individuals may recover but remain carriers of the disease. As noted earlier, disease transmission mechanisms depicted in the bottom part of diagram and observations shown along the top, have suggested certain study hypotheses involving disease sources and factors (*x*), transmission (*y*∼*x*), and spatiotemporal patterns (*y*). These hypotheses can be investigated by means of rigorous spatiotemporal analysis and laboratory testing. Cases and suspected determinant data were collected for this purpose.

### Spatial Statistics

Spatial modeling goes beyond standard data analysis by providing new dimensions that can process additional information, discriminate factors that may not be perceivable (in simple time series analysis for example), and locate sources and factors for accurate intervention. We examined clustered and geographically correlated cases, spatial patterns linked to suspected sources, and case distribution-disease determinant consistencies suggesting transmission mechanisms. These were analyzed by means of, local indicator of spatial association (LISA), spatial buffering, and geographical detector techniques [24] respectively. Multivariate analysis was performed by the interaction detector technique, measuring the spatial consistency between disease distribution and a distribution formed by overlaying the distributions of the multivariate of the determinants. Multivariate regression was not suitable because either the sample size was too small (9 towns in Hongta district) or the associated explanatory variables were not available (in villages and streets).

Local Moran's *I*, i.e. LISA measures the spatial autocorrelation of an attribute as [22]

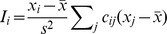
where *x_i_* is the attribute value in the spatial cell *i*; *c* is the matrix of spatial weights, e.g., *c_ij_* denotes the weight of relationship between cells *i* and *j*; 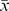 and *s* ^2^ denote the mean and variance of *x*, respectively. A positive *I_i_* implies that the cell *i* and its neighbors are similar; e.g., high-high or low-low cells. A negative *I_i_* implies that the cell *i* and its neighbors are dissimilar; e.g., high-low or low-high cells. LISA was performed using the publically available software GeoDA software.

An object's buffer refers to a zone covering a specified distance around it. For a single object a series of buffers may be considered at increasing distances. If the object is a point (e.g. a market), its buffers are a series of circles centered at that point. If the object is a line (e.g., a canal), its buffers are a series of belts parallel to the line. If the prevalence in the buffers exhibits a trend with distance from an object, it implies that the specified object is associated with disease transmission. Buffer statistics were calculated using ARCGIS software.

The geographical detector [24] is used to assess environmental risks to human population. The method is different from conventional mathematical correlation or regression, which require that the determinants or their proxies are quantitative. It is assumed that the disease will exhibit a spatial distribution similar to that of an environmental factor if the environment contributes to disease transmission, as measured by the Power of Determinant (*PD*),
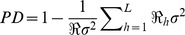
where *ℜ* and *σ* ^2^ denote the area and the dispersion variance of disease incidence of the study area, respectively. The study area is stratified into *L* stratums, *h* = 1, …, *L* [23] according to spatial heterogeneity of a suspected determinant or its proxy of the disease. Note that spatial heterogeneity is defined as an attribute whose statistical properties (e.g., mean and standard deviation) change in space. *PD* ∈[0, 1], where 1 indicates that the determinant completely controls the disease, and 0 indicates that the determinant is completely unrelated to the disease. In other words, *PD* expresses the extent to which a determinant explains disease incidence. In addition, disease transmission is often determined by multiple factors. The *PD*, combined with spatial overlay techniques of GIS and set theory, yields a geographical detector that can effectively assess the relationship between multiple factors: Are these factors independent or interacting? Are they enhancing or weakening each other? Is their interaction linear or nonlinear? In the present study, the geographical detector was implemented using the software www.sssampling.org/geogdetector (publicly available).

## Results

### Spatiotemporal Distribution

**Figure 3:**
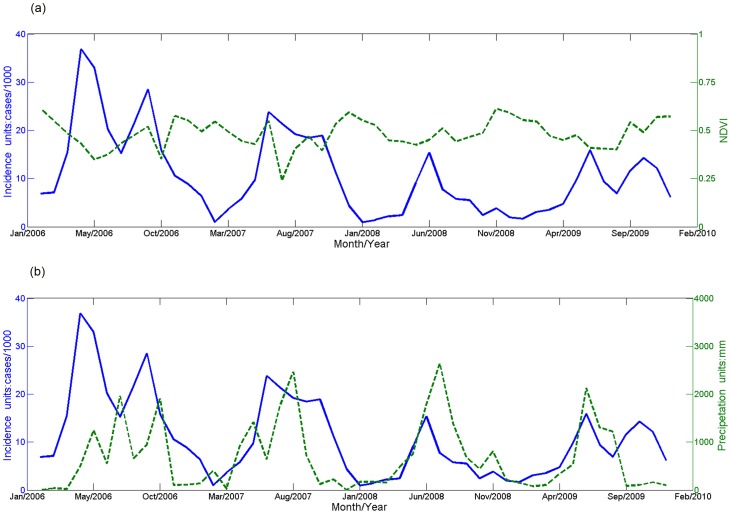
The temporal variability of typhoid and paratyphoid incidence and relevant factors in Hongta district. (a) Relationship between incidence of typhoid and paratyphoid and NDVI; (b) Relationship between incidence of typhoid and paratyphoid and precipitation.

The overall disease distribution as a function of time is shown in Table 1. Figure 3 shows that the disease incidence is in inverse phase with NDVI (Figure 3a) and in the same phase as precipitation (Figure 3b).

**Table 1:** Typhoid and paratyphoid distribution by age group and by month of disease onset.

Characteristics	Number of cases	Incidence (cases/100,000)
Age (years)		
0∼4	10	3.2
5∼9	28	10.6
10∼19	194	30.3
20∼29	413	48.5
30∼39	330	42.8
40∼49	174	38.6
50∼59	107	30.3
60∼	51	11.9
Month of onset		
Jan	39	9.6
Feb	49	12.5
Mar	89	21.9
Apr	201	49.4
May	181	44.6
Jun	115	28.2
Jul	100	24.6
Aug	131	32.2
Sep	182	44.7
Oct	104	25.6
Nov	71	17.5
Dec	45	11.1

**Figure 4:**
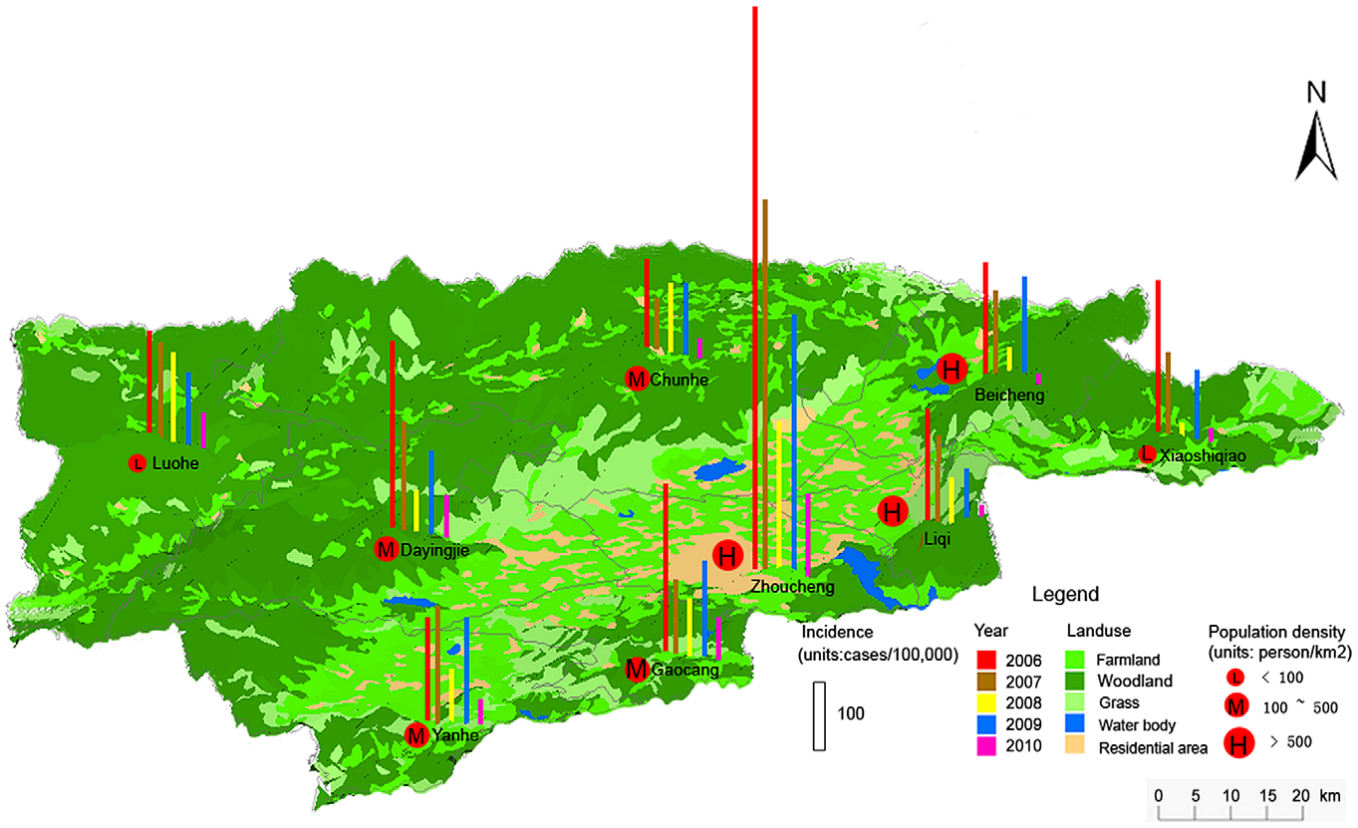
Yearly incidence of typhoid and paratyphoid in nine towns, Hongta district. Landuse as categorized by farmland, woodland, grass, water bodies and residential areas are mapped using different colors; red circles marked by L, M and H denote towns with low, medium and high population densities respectively.

**Figure 5:**
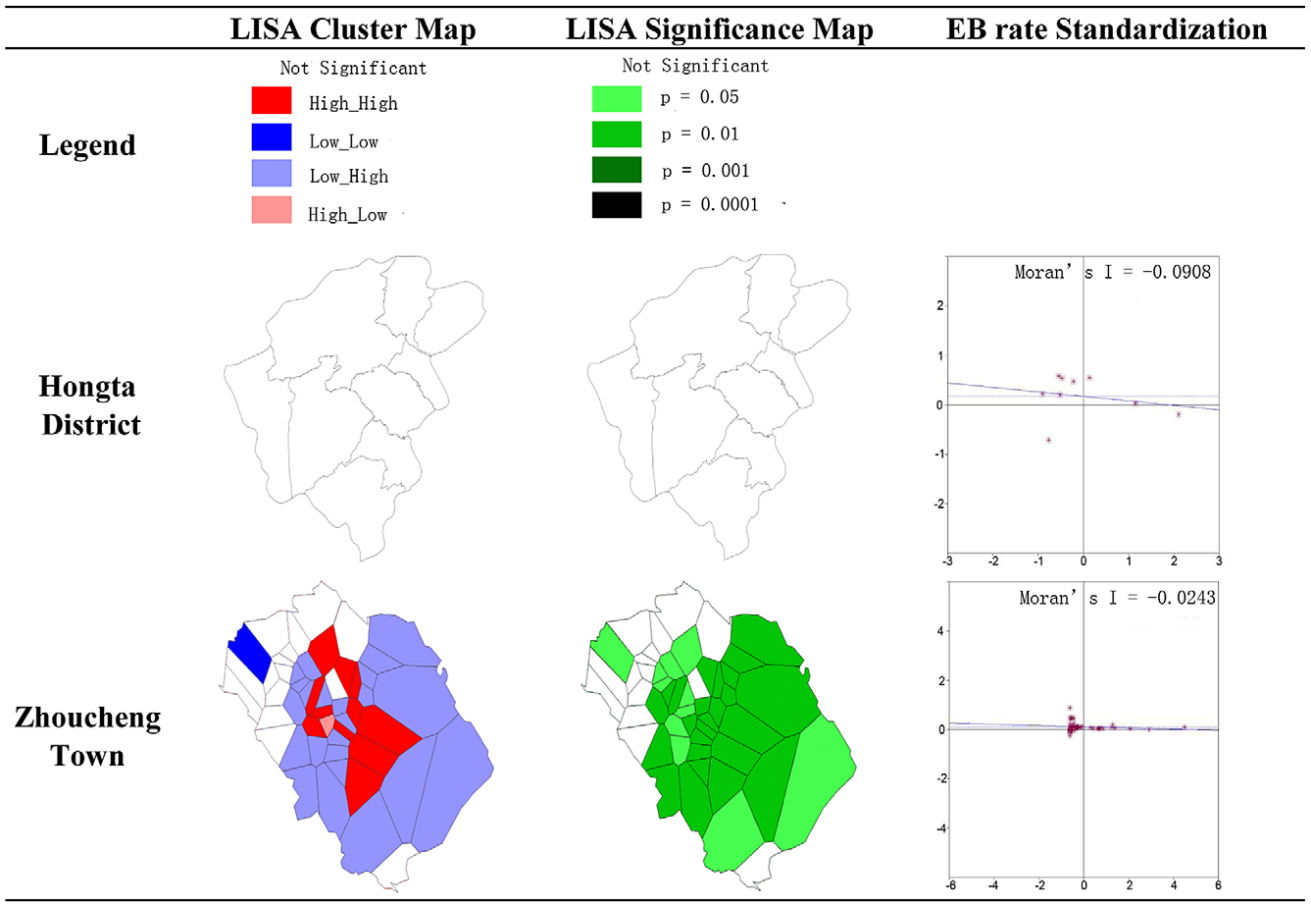
LISA in Hongta district and Zhoucheng town.

Figure 4 displays yearly incidence in 9 towns of the Hongta district during the period 2006–2010, with the exception of 2009, together with population densities and landuse. Towns are categorized as having low (L), medium (M) and high (H) population densities. Zhoucheng town is located at the south-eastern and central area of Hongta district and is made up of 51 villages. It has the highest population density and much higher incidence than towns in the western and northern hill woodland, which is why it was made the focus of this study. LISA statistic (P = 0.01) in Figure 5, shows that low incidence villages are close to other low incidence villages in the outskirts, whereas villages forming high incidence clusters are located close to the town centre. Higher disease incidence occurs in areas with a larger proportion of farmers in the population. Data relating to distribution of canals and markets are also available.

**Figure 6:**
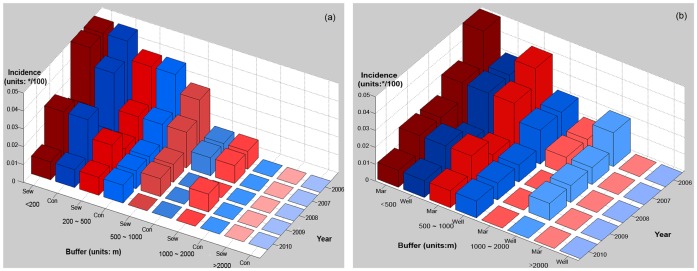
The spatial variability of typhoid and paratyphoid incidence and suspected determinants in the Zhoucheng town. (a) Relationship between incidence of typhoid and paratyphoid and sewer/confluence; (b) Relationship between incidence of typhoid and paratyphoid and market/well (Sew denotes Sewer; Con denotes Confluence; Mar denotes Market).

Figure 6 displays the associations between disease incidence and suspected determinants in Zhoucheng town. The x-axis represents the distance between sewer and confluence (in Figure 6a) and between market and well (in Figure 6b), shown with their respective buffer zones. The y-axis denotes years (2006–2010), and the z-axis the population incidence. Figure 6a shows that the population incidence increases with decreasing distance from the canals. Interestingly, the incidence reduces rapidly away from confluence canals (note: blue bars rapidly shorten along buffer-axis), whereas it reduces rather slowly away from sewage canals (red bars slowly shorten along buffer-axis).Figure 6b shows that disease incidence reduces with distance from markets and groundwater wells, the decline being more rapid from the markets (red bars rapidly shorten along buffer-axis) than from the wells (blue bars slowly shorten along buffer-axis). Figures 6a and b, show that the abnormally high incidence during 2009 occurred within a 500 m buffer zone of sewers and confluences and an 800 m buffer zone of markets and wells, suggesting that the suspected disease sources are not only a cause of the observed spatial disease distribution but they were also responsible for the 2009 disease outbreak.

After being standardized as (*y*−min)/(max−min), the time series of incidence, precipitation and NDVI were analyzed. The typhoid fever and the paratyphoid fever both fluctuated in time, experiencing two peaks per year; the first peak from April to June and the second from September to November. The incidence was positively correlated with the precipitation time series and usually peaked about one month earlier or one month later than the precipitation peak. NDVI is an index reflecting biological mass, and its time series appears to be negatively correlated with disease incidence.

### Multivariate Interaction

Laboratory testing indicated that the *Salmonella* strains found in water samples from hospitals, canals, farmland, and market vegetables belonged to closely related clones of *S. typhi*. Vegetables sold in the markets came from farmland irrigated by water contaminated by hospital and residential sewage. The findings of our spatiotemporal analysis, that hospital and residential sewage discharge and food markets are the most probable routes of disease transmission, support previous surveys carried out in this area.

In addition to these direct disease sources, other environmental factors are believed to exert an influence on disease transmission. These include factors such as economic and social status, types of disease intervention, climate-biology types and physical conditions. However, these factors are rather difficult to measure in a straightforward manner. Instead, they are represented by their proxies: GDP per capita (economic determinant), proportion of farmers in total population (social determinant), number of medical institutions (intervention), NDVI (climate-biology type), and geomorphic types (environmental conditions).

Using the geographical detectors it was found that social factors have the highest disease impact of all those measured (Table 2). The combined impact of two factors *A* and *B* on disease incidence generally differs from the simple linear summation of the separate *A* and *B* impacts, i.e., *A* ∩ *B*≠*A*+*B*. The spatial incidence distribution is highly correlated with that of farmer proportion and population density. The proportions of spatial prevalence variation explained by these two factors reach *PD* = 78.3% (for *A*) and 73.8% (for *B*). The farmer proportion and population density together explain 84.7% of the variation of disease incidence, higher than that explained by any single factor.

**Table 2:** Interaction between factors affecting the prevalence of typhoid and paratyphoid.

*A* 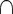 *B* = *C* (%)	*A* (%)	*B* (%)	*A*+*B* (%)	Conclusion	Interpretation
GDP per capita 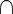 NDVI = 23.4	8.7	1.8	10.5	*C*>*A*+*B*; *C*>*A*, *B*	*A* enhances *B*
GDP per capita 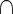 Num. of medical inst. = 53.8	8.7	18.7	27.4	*C*>*A*+*B*; *C*>*A*, *B*	*A* enhances *B*
Farmer proportion 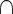 Population density = 84.7	78.3	73.8	152.1	*C*<*A*+*B*	*A* weakens *B*
Geomorphy 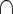 Population density = 79.4	20.1	73.8	93.9	*C*<*A*+*B*	*A* weakens *B*
Geomorphy 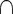 GDP per capita = 28.9	20.1	8.7	28.8	*C = A*+*B*	*A* independent of *B*

Note: *A* and *B* denote two determinants, with their *PD* values in geographical detector. The *PD* value measures the % of spatial disease variance explained by a determinant; *A*∩*B* refers to a map formed by an overlay by *A* and *B*; *C* is the *PD* value of the *A* and *B* overlaid map. For more detail of geographical detector please refer to the main text or literature [24].

The incidence is weakly associated with the economic condition (GDP per capita) and the climate-biological type (NDVI), with *PD* values of 9.7% and 1.8%, respectively. However, the incidence is more strongly linked with the spatial combination of GDP and NDVI. More than half (53.8%) of the spatial variation of the incidence can be explained by the spatial distribution of economic condition (GDP per capita) combined with availability of medical intervention (number of medical institutions). The individual determinant impact of GDP and NDVI are clearly low (8.7% and 18.7% respectively). Geomorphology explains only a small part of spatial disease transmission, but its impact increases when it is combined with population density or GDP per capita. These findings jointly imply that a policy instrument based on analysis of a combination of the most relevant determinants,(in this case, population density and farmer proportion), would be highly efficient in controlling spatial disease transmission in a region.

## Discussion

Based on the results of rigorous spatiotemporal analysis and GIS technology, disease transmission processes can be identified, modeled, and used for population health management purposes. The findings of the present Hongta district study include the following: Unsterilized water from hospital disposal and residential sewage are discharged into the rainwater canal system to irrigate the region's farmlands where local vegetables are grown. Polluted vegetables are sold in the markets and consumed without being adequately washed. Infection is magnified during the rainy seasons, when more contaminated water irrigates the farmlands and the polluted water, mixed with additional garbage spreads over a larger area of the inhabited region. Over time, the combination of the farmers' poor hygiene conditions and low cure rates lead to considerable pathogen reserves among the population. The general seasonal-intestinal infection cycle, driven mainly by annual temperature fluctuations and varying volume of polluted canal water in the irrigation system driven mainly by the rainy seasons, combine to cause the persistent high endemic transmission of the disease in the Hongta district.

The availability of spatiotemporal data and the development of health information systems facilitate the GIS-spatiotemporal exploration of public health issues. The physical and social determinants of health are usually spatially distributed: surface and subsurface contaminated water, polluted water emission from workplaces, disease prevention methods, and nutrition and food habits. More importantly, the susceptible and the infected populations are always geographically distributed. Health determinants may be detected when the disease incidence shares similar spatial features with environmental features suspected of being associated with the disease. When disease cases and environmental features do not share similar spatial features, however, this does not necessarily mean that they are unrelated. Instead, the geographically dispersed disease cases may have the same casual sources as features which may be elucidated by alternative techniques such as molecular tracking in food or serum samples, and food electronic label tracking systems.

This study identified some of the disease sources, determinants, and transmission cycles, which affect the distribution of typhoid and paratyphoid cases in the Hongta district which currently exhibits the highest incidence in China. This knowledge can be used to design intervention measures to reduce and hopefully eradicate the disease in this area. In addition to the measures common to the control of enteric infectious disease intervention, such as washing hands before eating and environmental sanitation, particular attention should be paid to the sterilization of hospital waste prior to discharge into canals, especially during the rainy season.

## Supporting Information

Checklist S1 STROBE Checklist.
(PDF)
