# Correction: Spatiotemporal Transmission and Determinants of Typhoid and Paratyphoid Fever in Hongta District, Yunnan Province, China

**DOI:** 10.1371/annotation/f4d7cc29-9ea5-46cf-ad41-17728176df6d

**Published:** 2013-03-18

**Authors:** Jin-Feng Wang, Yan Wang, Jing Zhang, George Christakos, Jun-Ling Sun, Xin Liu, Lin Lu, Xiao-Qing Fu, Yu-Qiong Shi, Xue-Mei Li

In the Methods section, sub-section Spatial Statistics, the equation measuring the Power of Determinant (PD) contains an error. An italicized subscript 'h' should follow the final sigma symbol below the '2'

